# Concurrent lymphoma and hemophilia B in a pediatric patient: A case report

**DOI:** 10.1097/MD.0000000000015474

**Published:** 2019-05-13

**Authors:** Guoyan Lu, Lina Qiao, Deyuan Li, Zhongqiang Liu, Fumin Zhao, Dan Yu

**Affiliations:** a Department of Pediatric, West China Second University Hospital, Sichuan University, Chengdu; b Key Laboratory of Birth Defects and Related Diseases of Women and Children (Sichuan University), Ministry of Education, Chengdu, Sichuan; c Department of Radiology, West China Second University Hospital, Sichuan University, Chengdu, China.

**Keywords:** cancer, child, hemophilia, lymphoma, non-Hodgkin

## Abstract

**Introduction:**

Lymphoma is the third most common cancer among children in the United States and Europe. Hemophilia is a congenital bleeding disorder characterized by deficiency of coagulation factor VIII or IX. Hemophilia B is a consequence of factor IX deficiency and has an incidence of 1 in 20,000 male births. A concurrence of these 2 uncommon diseases is rare except in patients infected with the human immunodeficiency virus (HIV). We report a case of a patient with both Burkitt lymphoma and hemophilia B; this is only such report in China since 1987.

**Patient concerns:**

A 3-year-old boy was admitted to our hospital because of melena and jaundice for several days. His older brother had died due to hemophilia B and ventricular septal defect. The patient had not experienced any previous episodes of severe bleeding. Gradual abdominal distention was observed after admission; the patient's superficial lymph nodes were not enlarged. Results of blood routine and bone marrow examinations showed no abnormalities. He was diagnosed with sclerosing cholangitis, abdominal infection, and hepatitis. However, after treatment of reducing enzyme activity and eliminating jaundice, the patient's condition deteriorated. Hydrops abdominis was detected on abdominal ultrasonography. Tumor cells were found by pathological examination of peritoneal effusion. Both a c-myc gene translocation and a c-myc-IgH gene fusion were detected.

**Diagnosis:**

Burkitt lymphoma and hemophilia B.

**Interventions:**

The patient was transferred to the Pediatric Hematology Department of our hospital and treated with a modified B-NHL-BFM-95 protocol. During chemotherapy, platelet changes were monitored regularly and blood products were infused timely.

**Outcomes:**

The patient died of infection and bleeding after chemotherapy.

**Conclusion:**

Concurrent hemophilia and lymphoma are rare, especially in children. When encountering a patient with unexplained obstructive jaundice and massive ascites, the possibility of a tumor should be considered. Early diagnosis and adequate treatment of such tumor may improve prognosis.

## 1 Introduction

Lymphoma is the third most common cancer among children in the United States and Europe.^[1–4]^ Hemophilia is a congenital bleeding disorder characterized by deficiency in the coagulation factors VIII or IX; of all cases, 85% and 15% occur due to factor VIII and IX deficiency, respectively.^[5]^ Simultaneous onset of the 2 diseases is rare except in patients infected with the HIV.^[6–9]^

Here, we report a case of simultaneous onset of Burkitt lymphoma and hemophilia B in a HIV-negative boy. This is only the second report of concurrent hemophilia and lymphoma in China since 1987.

## 2 Ethics statement

Informed consent was obtained from the patient's parents for publication of this case report and the accompanying images.

## 3 Case report

A 3-year-old boy presenting with melena and jaundice for several days was admitted to our Pediatric Gastroenterology Department. His anthropometric measurements were appropriate for his age. Pallor, scleral icterus, petechiae, and ecchymoses on his legs were observed. Systemic examination revealed no lymphadenopathy, hepatomegaly, or splenomegaly. Peripheral blood counts revealed a hemoglobin level of 87 g/L, white blood cell count of 7.1 × 10^9^/L, and platelet count of 484 × 10^9^/L. A coagulation test showed the following results: prothrombin time, normal; activated partial thromboplastin time, markedly prolonged (131.1 seconds; normal range: 20.4–40.4 seconds); and plasma factor IX clotting activity level, 2.7% (normal range: 70%–120%). The patient's older brother had died of hemophilia B and ventricular septal defect several years ago. Based on these results, the patient was diagnosed with hemophilia B. However, he had no serious bleeding tendency and had never received a blood transfusion or any infusion of coagulation factors before admission. After admission to our hospital, his bleeding was stopped by transfusion of coagulation factors.

Because the patient presented with jaundice, we performed a series of tests. A liver function test revealed the following levels: total bilirubin, 72.9 μmol/L (normal range: 3–22 μmol/L); direct bilirubin, 66.4 μmol/L (normal range: <5 μmol/L); alanine aminotransferase, 192 U/L (normal range: <50 U/L); aspartate transaminase, 168 U/L (normal range: <50 U/L); γ-glutamyl transferase 791 U/L (normal range: <70 U/L); lactate dehydrogenase, 2,629 U/L (normal range: 300–600 IU/L); and albumin, 32 g/L (normal range: 35–50 g/L). A HIV serological test was negative. Other tests, including those for hepatitis markers, TORCH (toxoplasma, rubella virus, cytomegalovirus, and herpes simplex virus), serum ceruloplasmin, as well as Epstein–Barr virus nucleic acid and autoantibody indicated normal results.

**Figure 1:**
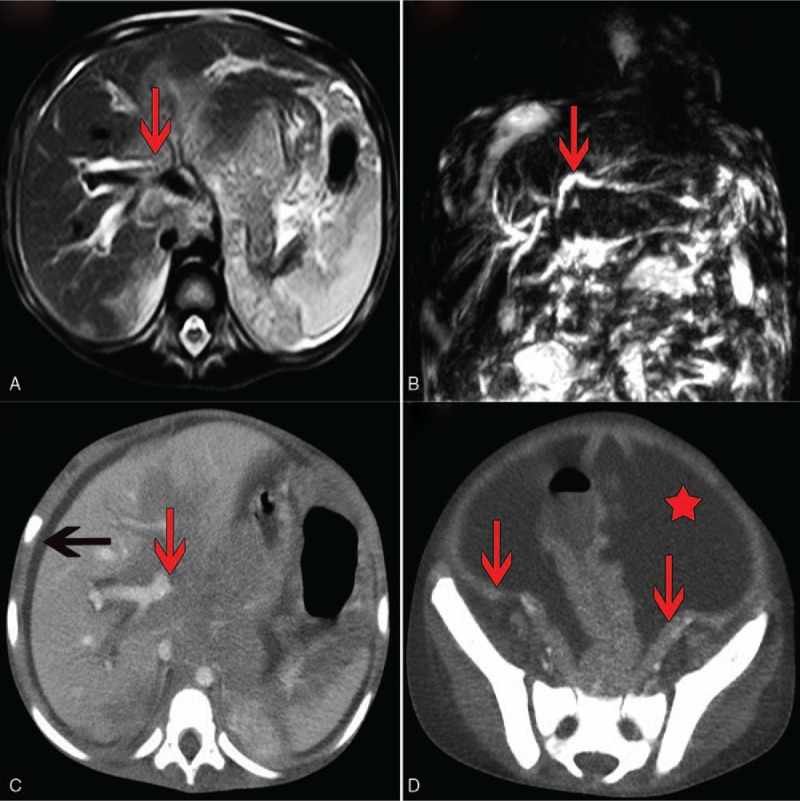
Imaging results magnetic resonance cholangiopancreatography (MRCP) showed lymph node enlargement and fusion in the hilar region, with a slightly high-density shadow (A) as well as mild dilatation of the intrahepatic bile duct (red arrow), no expansion of the extrahepatic bile ducts, and no manifestation of the gallbladder or common bile duct (B). Abdominal contrast-enhanced computed tomography (CT) showed enlargement of lymph nodes in the hilar region (red arrow) and perihepatic effusion (black arrow) (C) as well as a large amount of ascites (red asterisk) and diffuse incrassation in the peritoneum (red arrow) (D).

After admission, we observed gradual abdominal expansion and hepatomegaly. An abdominal ultrasound indicated hydrops abdominis. Multiple blood routines and bone marrow examinations revealed no abnormalities. Magnetic resonance cholangiopancreatography (MRCP) (Fig. 1A, B) showed enlarged lymph nodes in the hilar region, in the areas of the hepatic ligament and round ligament, and in the space of the portal cavity. The lymph node enlargements resulted in hepatic hilar biliary duct obstruction and intrahepatic biliary dilation. Extensive peritoneal thickness and a small amount of ascites were observed. The patient was diagnosed with sclerosing cholangitis, abdominal infection, and hepatitis. It was also necessary to exclude cancer. None of the lymph nodes were superficially enlarged; we consulted the surgeon regarding whether biopsy of the abdominal lymph nodes could be performed. Considering the hemophilia, the surgeon advised against lymph node biopsy because the abdominal lymph nodes were small and as the patient was thought to have a high risk of bleeding.

**Figure 2:**
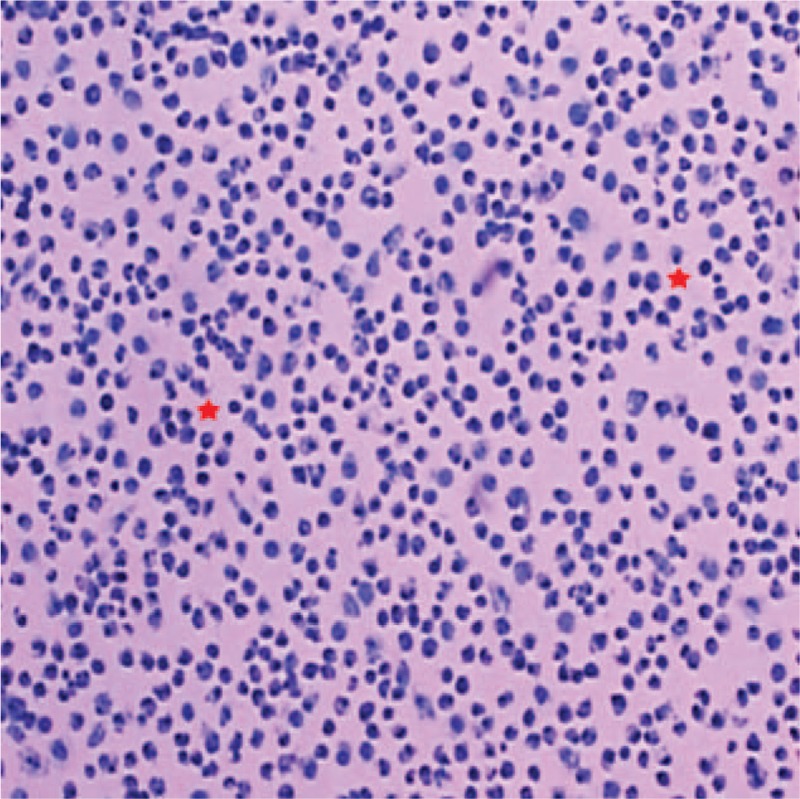
Hematoxylin and eosin stain (original magnification × 400). The image depicts a high number of atypical lymphoid cells (red asterisk) and substantial apoptotic debris.

We attempted treatment to protect the patient's liver by reducing enzyme activity, resolving jaundice, and providing anti-inflammatory and supporting therapy. However, all measures proved ineffectual and the patient's condition deteriorated. We found that the ascites had increased gradually. Abdominal contrast-enhanced computed tomography (CT) (Fig. 1C, D) on day 20 after admission showed a large amount of ascites, some intumescent lymph nodes with abnormal density around the hepatic hilar region or at the root of the mesentery, and diffuse incrassation in the peritoneum and mesentery. After obtaining parental consent, we decided to risk performing abdominal puncture. Tumor cells were found in the ascites (Fig. 2). Flow cytometry of the ascites showed B lymphocyte-group cells with restricted light chain expression; these results were considered to indicate lymphoma. Using fluorescence in situ hybridization, both a c-myc gene translocation and a c-myc-IgH gene fusion were detected. Finally, the patient was diagnosed with Burkitt lymphoma, a type of non-Hodgkin lymphoma. He was transferred to the Pediatric Hematology Department of our hospital and treated with a modified B-NHL-BFM-95 protocol. Because of the patient's poor physical condition and malnutrition, he did not tolerate high-intensity chemotherapy. After 3 cycles of chemotherapy, he died from severe infection and bleeding.

## 4 Discussion

Factor IX deficiency causes hemophilia B; this X-linked disorder has an incidence of 1 in 20,000 male births.^[10]^ Patients are unable to generate adequate thrombin and stable fibrin during blood coagulation and therefore have a bleeding diathesis. Patients with hemophilia are considered a high-risk population for hematological malignancies, in particular Burkitt lymphoma. ^[11,12]^ Malignant tumors may occur in such patients in the setting of acquired immunodeficiency syndrome (AIDS)^[6–9]^ Furthermore, it has been observed that patients with hemophilia have long-term immune function abnormalities due to factor concentrate usage.^[13–15]^ Thus, immunological dysfunction caused by factor concentrate usage and/or HIV infection are factors that likely contribute to a high risk of lymphocytic malignancy. In the present case, the patient had not received any infusion of coagulation factors or blood transfusion before admission. Furthermore, the patient was HIV-negative. Therefore, lymphoma development was not associated with AIDS. Environmental factors can also lead to increased cancer risk. One of the most famous carcinogenic associations is exposure to ionizing radiation.^[12,16]^ An increased risk of malignancy related to diagnostic imaging has been described among both pediatric and adult patients.^[17–19]^ Patients with hemophilia may undergo diagnostic imaging more frequently to assess bleeding or joint diseases; however, the child in this case never experienced any severe episodes of bleeding and had never undergone multiple radiographic examinations. Therefore, it is likely that the association of the 2 disorders (lymphoma and hemophilia B) is merely accidental, although further confirmation is needed.

Burkitt lymphoma is a very aggressive B-cell non-Hodgkin lymphoma characterized by deregulation and translocation of the C-MYC gene from chromosome 8.^[20]^ Three clinical Burkitt lymphoma subtypes are recognized: endemic, sporadic, and associated with immunodeficiency. The endemic form (which is most frequent) mainly affects the facial bones, whereas the sporadic form mainly affects the terminal ileum, the cecum, and the intra-abdominal lymph nodes. The immunodeficiency-associated form is seen in HIV-infected patients, patients with autoimmune diseases, and those with primary immunodeficiency disorders.^[21,22]^ Obstructive jaundice is an unusual manifestation of pediatric non-Hodgkin lymphoma.^[19,23]^ Biliary obstruction and jaundice may be due to the compression caused by lymph node enlargement. Furthermore, the ascites may be caused by portal hypertension due to the compression caused by enlarged lymph nodes in the hepatic hilar region.

Due to the aggressiveness of Burkitt lymphoma, the disease is generally treated with intensive chemotherapy regimens. Early diagnosis and treatment allow for good patient survival.^[24]^ The survival rate of 10-year-old children diagnosed between 2005 and 2009 was reported as 90.6% in the United States. ^[24]^ Concurrent lymphoma and hemophilia pose a challenge to the development of therapeutic strategies; this is especially true when a patient with hemophilia presents only with jaundice and melena, but without lymphadenopathy or hepatosplenomegaly. Thrombocytopenia is a complication associated with chemotherapy; it can aggravate a bleeding tendency in patients with severe hemophilia and may cause a life-threatening bleeding event.^[5]^ During chemotherapy, it is therefore important to monitor platelet counts and reduce the risk of bleeding. At the time of the final diagnosis, the patient was unable to tolerate chemotherapy due to his poor physical condition and malnutrition. He eventually died of infection and bleeding.

Because disease progression may be gradual and disease symptoms are unspecific, early diagnosis is difficult in lymphoma. Several lessons can be learnt from the case presented herein: the superficial lymph nodes of the patient were not enlarged. Neither hepatomegaly nor splenomegaly was observed at the beginning of the disease. If routine treatment such as anti-infective therapy or anti-inflammatory was ineffective, the possibility of a tumor should be considered, especially if a patient has massive ascites and obstructive jaundice. The illness was initially considered to be a severe infection and sclerosing cholangitis. The use of steroids may have masked the actual condition(s). The surgeon did not perform a biopsy of the abdominal lymph nodes by exploratory laparotomy owing to the patient's hemophilia. If a biopsy had been performed, an early diagnosis may have been possible.

## 5 Conclusion

Concurrent hemophilia and lymphoma are rare, especially in children. This is only such report of concurrent hemophilia and lymphoma in China since 1987. When unexplained obstructive jaundice and ascites occur, the possibility of tumor disease should be considered and ruled out because early diagnosis and appropriate treatment of the tumor may improve prognosis.

## Author contributions

**Data curation:** Fumin Zhao.

**Formal analysis:** Zhongqiang Liu.

**Funding acquisition:** Dan Yu.

**Methodology:** Zhongqiang Liu.

**Project administration:** Deyuan Li.

**Resources:** Dan Yu.

**Software:** Fumin Zhao.

**Supervision:** Lina Qiao.

**Writing – original draft:** Guoyan Lu.

**Writing – review and editing:** Dan Yu.
